# Protocol for the home hazards removal program (HARP) study: a pragmatic, randomized clinical trial and implementation study

**DOI:** 10.1186/s12877-017-0478-4

**Published:** 2017-04-20

**Authors:** Susan Stark, Emily Somerville, Marian Keglovits, Jane Conte, Melody Li, Yi-Ling Hu, Yan Yan

**Affiliations:** 1 Program in Occupational Therapy, Washington University School of Medicine, 4444 Forest Park Avenue, Campus Box 8505, St. Louis, MO 63108 USA; 2 Division of Biostatistics, Washington University School of Medicine, 660 South Euclid Avenue, Campus Box 8067, St. Louis, MO 63110 USA

**Keywords:** Older adults, Falls, Home hazards, Medically underserved, Randomized controlled trial, Implementation trial

## Abstract

**Background:**

Falls remain the leading cause of injury, long-term disability, premature institutionalization, and injury-related mortality in the older adult population. Home modifications, when delivered by occupational therapists, can reduce falls among high-risk community-dwelling older adults by 39%. However, home-modification implementation is not standard practice in the United States. The goal of the *H*ome H*a*zard *R*emoval *P*rogram (HARP) study is to implement an evidence-based home modification intervention for older adults designed to reduce the incidence of falls through an aging services network.

**Methods:**

We will conduct a hybrid effectiveness/implementation trial of 300 older adults at risk for a fall who are randomized and followed for 12 months. Participants who are randomized to treatment will receive the home modification intervention provided by an occupational therapist in addition to usual care, defined as continued services from the area agency on aging. We will compare the effectiveness of the program and usual care using survival analysis with the time to the first fall over 12 months as the primary outcome of interest. Secondary outcomes include daily activity performance, fall self-efficacy, and health-related quality of life. Fidelity, dose, adherence, safety, cost, and health care utilization will also be examined in the implementation component of this study.

**Discussion:**

This intervention targets an underserved, difficult to reach population of older adults. The tailored approach of the study intervention is a strength in improving adherence, as each recommendation is individualized to be acceptable to the participant. The effectiveness/implementation design of the study allows for rapid dissemination of results and implementation of the intervention in a United States social services agency.

**Trial registration:**

Clinicaltrials.gov identifier: NCT02392013. Retrospectively registered on March 5, 2015.

## Background

Falls remain the leading cause of injury, long-term disability, premature institutionalization, and injury-related mortality in the older adult population [1–4]. Falls are the most common cause of traumatic brain injury and fracture for older adults [5], and they have serious complications such as institutionalization [6], functional dependence, paralyzing fear of falling, and death [7–9]. Falls are an eminent threat to a frail older adult’s ability to maintain independence in the community. Approximately 1 in 3 community-dwelling adults aged 65 years and older fall each year [10, 11], and those older than age 70 have an especially high fall risk [3]. Older adults who have experienced a previous fall are at a greater risk of falling again [12]. The majority of falls experienced by older adults, particularly more frail, high-risk older adults, occur in the home [1, 13, 14], and measurable home hazards are associated with an increased risk of older persons falling in the US [15]. Falls are costly: $30 billion a year is spent treating older adults for the effects of falls [16]. As the population ages, costs associated with falls are projected to reach $59.6 billion by 2020 [11], making fall prevention a public health priority.

Home modifications include installation of stairway railings, grab bars, slip-resistant surfacing in the bathroom, and provision of lighting [17]. Efficacy studies conducted in Australia and Europe and results from our own efficacy trial suggest that home modifications, *when delivered by occupational therapists* (OTs), can reduce falls among high-risk community-dwelling older adults by 39% [17–20]. Despite these findings and recommendations from the American Geriatrics Society that home modifications and home hazard removal be routine for community-dwelling older adults at high risk for a fall [21], home-modification implementation is not standard practice in the US. New models of care must be developed to effectively implement this strategy nationally.

Home modifications to improve daily activity performance have been studied in the US [22, 23], and there is promising preliminary evidence of the *efficacy* of home hazard removal to reduce the risk of falls (including our unpublished trial). However, no evidence shows the pragmatic *effectiveness* of home modifications to prevent falls in the US. To our knowledge, this trial represents the first time an effective home modification intervention to prevent falls will be implemented through a US social service delivery system, a regional area agency on aging (AAA). Community-engaged research approaches [24–27] will be used to determine adaptations necessary to deploy a home modification intervention to prevent falls in the US within a reliable aging services network. We will reduce barriers to program participation by involving key stakeholders as part of the research team [28, 29] and clarifying the benefits of the research to the community [30].

The *H*ome H*a*zard *R*emoval *P*rogram (HARP) study is a hybrid effectiveness/implementation trial of 300 older adults at high risk for a fall, along with a process evaluation [31] of the intervention to aid in dissemination and interpretability of the trial by evaluating acceptability, feasibility, safety, and cost. We hypothesize that older adults who receive the program will have a lower rate and risk of falls and improved self-efficacy, daily activity performance, and quality of life compared with the usual care group. We also predict that home modification interventions will have high acceptability (80% retention), high fidelity by therapists (95% of elements; 90% of dose delivered), low safety risk (no increased rate of falls compared with the usual care group), and high adherence (80% of modifications in use) at 12 months.

## Methods/design

### Research design overview

**Fig. 1:**
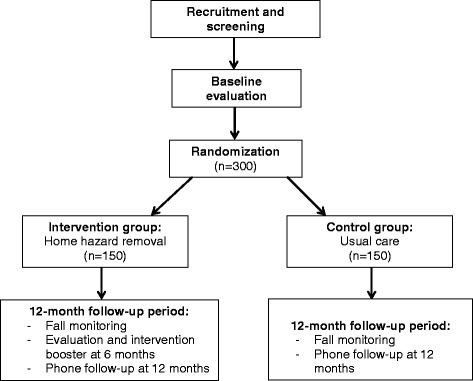
Flow diagram showing design overview for HARP study

To examine the effect of a home modification (home hazard removal) program, we will conduct a hybrid effectiveness/implementation trial of 300 older adults at risk for a fall who will be randomized to a home hazard removal program or usual care and then followed for 12 months. Fig. 1 outlines the flow of events for this study.

### Setting

This intervention will be implemented and evaluated in the urban core of St. Louis, Missouri, an area of high need for an effective fall prevention program. In Missouri, the fall death rate for older adults is consistently higher than the national average. The rate nearly doubled between 2000 and 2009 from 38 to 72.32 per 100,000 older adults [32]. Falls are the leading cause of unintentional injury-related hospitalizations and emergency department visits among older Missourians; older adults account for 64% of all ED visits and hospitalizations resulting from falls, a rate that is higher than national rates [33, 34].

### Study participants

We will recruit from a cohort of older adults who are “at high risk for a fall” [35]. The inclusion criteria are as follows: 1) aged 65 years or older; 2) self-report of one or more previous falls in the preceding 12 months or self-report as “worried about falling;” and 3) currently receiving services from St. Louis Area Agency on Aging (SLAAA). The exclusion criteria are as follows: 1) residents of nursing homes; and 2) individuals with severe cognitive impairment who are unable to give consent to participate (as determined by a score of greater than 10 on the Short Blessed Test) [36].

### Recruitment

We will recruit participants by utilizing services provided by St. Louis Area Agency on Aging (SLAAA) and the National Aging Program Information System (NAPIS). SLAAA offers unprecedented access to a population of older adults who are difficult to reach in the traditional health care system and could benefit from the intervention. The NAPIS is a database of information collected annually by the AAA describing the general health, nutritional, financial, functional, and environmental status of an older adult. In the current cohort of 1331 older adults screened in 2013 by SLAAA, the average age of the cohort is 77 years, 67% are female and 74% are African American. More than 25% reported a fall in the past 12 months, and 40% are “worried about falling.” The average score on performance of activities of daily living (ADLs) was 4 (range 1–7), indicating moderate ADL impairment). We estimate more than 800 older adults will meet eligibility criteria in the cohort [37, 38].

We will receive referrals from the older adults assessed annually by SLAAA via NAPIS who have reported fallen in the past 12 months or are worried about falling, and invite them to participate by telephone. SLAAA will also distribute our study flyer to recipients of home delivered meals to generate calls from interested older adults who are likely homebound and at risk for falling. Interested older adults will be screened for eligibility.

### Randomization and blinding

Upon signed consent, a study interventionist will elicit randomization. Participants will be allocated using a 1:1 ratio via randomization sequences generated a priori using a computerized formal probability model. Groups will be balanced with regard to race (self-reported African American or “other”) and sex. Randomization sequence concealment will be achieved by the Research Electronic Data Capture (REDCap) system [39]. Outcome raters and data analysis staff will be blinded to group allocation. However, the nature of the study does not allow for study interventionists and participants to be blinded to group allocation.

### Intervention

During the treatment phase of the study, each participant randomized to the control group will receive usual care from SLAAA, which includes an annual in-home NAPIS evaluation and individualized referrals to services based on identified need.

Participants who are randomized to treatment will receive the home modification (home hazard removal) intervention in addition to usual care. The intervention, based on a competence/press theoretical framework [40], will be provided by registered and licensed OT. Home modifications include changes to the physical environment (both spaces and objects within the environment), education about the physical environment and how to use it in a safer or more efficient way, and changing the social support to compensate for environmental barriers. A blend of all three approaches will be used in this intervention.

**Fig. 2:**
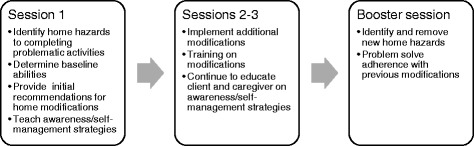
Overview of HARP intervention sessions

To ensure treatment fidelity, all participants will receive identical intervention components. During the initial evaluation, a 1-h home assessment is conducted by an OT in the participant’s home using the Westmead Home Safety Assessment (WeHSA) [41]. A comprehensive clinical assessment is also performed with each participant to determine functional limitations that may interact with hazards in the home and result in a fall. Environmental hazards and unsafe behaviors will be identified. A tailored home modification prescription is developed collaboratively during the first visit and assistance in removing the home hazards is provided by the occupational therapist in visits 1 through 3 (Fig. 2). Education about fall risks and self-management strategies to address fall risks are provided to participants throughout the intervention. A barrier removal plan will be provided to the older adult. The OT will facilitate obtaining home modifications, train the participant to use the recommended equipment and home modifications, and provide follow-up 2 weeks after the initial home visit to ensure that the participant adopted the recommended modifications. A booster session is provided 6 months following the intervention to identify and remove any new home hazards and identify/problem solve through any lack of adherence with previous modifications.

The three active components of this intervention are tailoring, self-management, and motivational enhancement. Home modifications are tailored to the participant’s needs and expressed wishes. Tailoring involves two key strategies: evaluating personal and environmental factors and shared decision making between the therapist and the participant. All aspects of the environmental intervention will be approved by the participant. Additionally, no part of the assessment or training will take place if the interventionist deems the activity or modification to be unsafe. Tailoring supports the next active component of self-management—the participant learns to make decisions and play an active part in the solution process, which will be ultimately carried out without the aid of an occupational therapist in the future. In self-management, individuals take an active role in their health, developing the capacity to define their own goals and create concrete plans to solve day-to-day problems [42]. The purpose of self-management in this intervention is to develop strategies to bring awareness to fall risks in everyday life, and to teach generalization of strategies. The third active component, motivational interviewing (MI), is a client-centered, semi-directive method of enhancing intrinsic motivation to change behavior by developing discrepancy and exploring and resolving ambivalence within the client [43]. MI recognizes and accepts that clients who need to make changes in their lives (in this case, removing fall hazards and minimizing fall risk behaviors) approach interventions at different levels of readiness to change their behavior.

### Data collection

#### Primary endpoint

We will capture the incidence of falls over a 12-month monitoring period. Prospective daily recording and surveillance of falls will be used at least once per month, as recommended by the ProFaNE consensus conference [44]. To record falls, an individually customized calendar journal will be designed for each participant for daily recording of the primary outcome (fall or no fall) [45]. Our method of tailoring the calendar journal includes life event date anchors, incentives, and personalization [46]. If a participant falls, they will be instructed to complete a fall form. Calendar journal pages and fall forms will be collected monthly via U.S. mail. Falls will be verified with a follow-up telephone interview by a community coordinator. Participants who are unable to complete the calendar journal (due to low vision, severe arthritis affecting writing, etc.) receive weekly phone calls from the study coordinator to ascertain falls.

#### Outcome measures

Important patient-reported outcomes [47] related to this intervention include daily activity performance, fall self-efficacy, and health-related quality of life. These will be assessed in the home at baseline and by phone at 12 months.

The Older Americans Resources and Services (OARS) ADL scale [48] will be used to screen for functional performance. Participants are asked about their ability to perform each of 14 activities (no help, some help, or unable to do). Responses are scored on a 0 to 2 scale, with higher scores indicating greater independence.

The Falls Efficacy Scale-International (FES-I) Short Form [49] will be used to assess participants’ self-efficacy in performing daily activities without falling. The FES-I has good test-retest reliability and validity and consists of a list of 7 daily activities (e.g., getting in and out of a chair) on which respondents rate their concern about falling while completing each of the daily activities with scores from 1 (no concern) to 4 (very concerned). Total FES-I score is the sum of each activity score, with higher scores indicating greater fear of falling.

Self-rated health will be measured using the 36-Item Short Form Survey (SF-36) [50]. The SF-36 is a commonly used tool to examine 8 dimensions of health including 1) physical functioning, 2) role limitation due to physical health problems, 3) bodily pain, 4) role limitation due to mental health problems, 5) energy/fatigue, 6) emotional well-being, 7) social functioning, and 8) general health perceptions. Each domain is scored from 0 (poor health) to 100 (optimal health).

#### Covariates of interest

Covariates of interest, including recognized risks for falling [51], will be collected at baseline, with the exception of the Westmead Home Safety Assessment (WeHSA), which will be collected at baseline and at the conclusion of the intervention in the treatment group. Demographic and other pertinent information, including assistance received, adaptive equipment usage, and falls, will be collected.

The Westmead Home Safety Assessment (WeHSA) [41] will be used to identify the number of environmental hazards in all areas of the home (e.g., seating, bedroom, medication management) via 72 categories. Each category is specified with explicit descriptors to qualify a given hazard with a score of 0 for absent and 1 for present. Total hazards are summed.

The Short Michigan Alcoholism Screening Test - Geriatric Version (S-MAST-G) [52] will be used to screen for the presence of an alcohol problem in participants. The Geriatric Depression Scale Short Form (GDS-SF) [53] will be used to assess depression levels in participants. The Short Blessed Test (SBT) [36] will be used to screen for severe cognitive impairment. The SBT has good validity for older adults [36] and correlates well with the Mini-Mental State Exam [54]. This brief, six-item questionnaire assesses cognition, memory, and orientation. Total scores range from 0 to 28; scores of 10 or more are consistent with dementia and other cognitive difficulties. Individuals who receive a SBT score 10 or more are not eligible to participate in the study.

A medication inventory will be collected to evaluate prescription medication use. Mobility and balance will be assessed using the Performance Oriented Mobility Assessment (POMA) [55], a task-oriented gait and balance assessment that has been validated for the older adult population. Range of motion and strength of the upper extremity will be assessed using group muscle tests and scored as within normal limits, within functional limits, or impaired [56].

We will measure visual contrast sensitivity using the Pelli-Robson test [57]. We will score participants’ binocular contrast sensitivity letter by letter. Protective behavioral factors to prevent falls will be assessed using the Falls Behavioral Scale for Older People (FaB) [58].

#### Implementation outcomes

##### Recruitment

Enrollment and retention will be tracked by the research coordinator using REDCap. Recruitment information for each participant will include potential participants’ demographic characteristics, enrollment status, and reason for decline.

##### Fidelity

In order to guarantee treatment fidelity, or our ability to provide the same treatment as planned to each participant, we follow similar methods of Weersing, et al. [59] and use a Visit-by-Visit Treatment Grid. This grid is a checklist of the pre-, during, and post-treatment visit requirements for each treatment session. During weekly interventionist meetings, the lead therapist will review the treatment grid for each participant to guarantee the necessary components of the intervention are being delivered.

##### Dose

In order to effectively measure the dose of treatment provided for each participant, we will measure both the dose that was delivered to each person (minutes of each treatment session and number of sessions) as well as the dose received (recommendations implemented/total recommendations). We will use a Time Log to track minutes spent in each treatment session and a Prescription Log to track the recommendations made and implemented for each participant.

##### Adherence

Adherence measures the participant’s continued use of the implemented modifications. We will calculate adherence using the standardized approach that Cumming, et al. [60] used: adherence = recommendations used/total recommendations. Interventionists will rate adherence with intervention at the final session by using the Prescription Log to track recommendations made, implemented, and reasons any recommendations were not implemented. Adherence will also be measured 6 months post-intervention. Reasons for abandoning strategies will be examined using the Adherence Log, in which the participant will report on current level of use for each modification: very useful, somewhat useful, not at all useful, or no longer use equipment. Any independence that was regained by improved sensorimotor performance will not detract from the adherence rating.

##### Safety

To evaluate the safety of our study, we will measure the number of falls and the circumstances surrounding the falls with a self-report Fall Form used at the 6-month follow up visit. The rate and severity of the falls will be calculated using a standardized algorithm established by Tinetti, et al. [1].

##### Cost

The cost of the treatment will be measured by tracking cost of modifications and adaptive equipment for each participant. This will be tracked using an Invoice Form which includes costs from the contractor as well as costs of any equipment ordered from a medical supply company or obtained from a community resource (e.g., medical equipment lending program).

##### Health care utilization

To determine health care utilization for each participant during the study, questions from the Stanford Chronic Disease Self-Management Study [61] will be asked at 6 and 12 months. These questions track number of emergency room and outpatient visits, number of hospitalizations, and number of days in therapy.

### Statistical analyses

We will compare the effectiveness of the intervention and usual care with the time to the first fall over 12 months as the primary outcome of interest. Survival analysis techniques will be used where time zero is the date on which the intervention is completed, and the ending time is 12 months after the intervention. Participants who do not have a fall will be censored at the end of the study. Participants who drop out of the study will be censored on the date participation concludes. Kaplan-Meier product limit estimates will be used to describe the fall experiences for the intervention and control groups, with the log-rank statistic testing for significant difference. Pre-specified covariates (e.g., age, race, sex, number of reported medications, mobility, ADL scores, and visual acuity) will be adjusted using the Cox proportional hazards model. We will test the interventions effect using the Cox proportional hazards model and adjusting for covariates. The regression coefficient of the indicator variable quantifies the difference in the log hazard of time to first fall between intervention and control groups. We will determine whether fall-prevention treatment is superior to usual care on secondary outcomes (total number of falls, number of injurious falls) and participant-reported outcomes (daily-activity performance, fall self-efficacy, and global health).

A data monitoring committee (DMC) will not be used due to the low risk of the proposed home modification intervention. Previous efficacy studies suggest that OT-delivered home modifications can reduce falls among high-risk community-dwelling older adults [17–20].

#### Sample size calculations

**Fig. 3:**
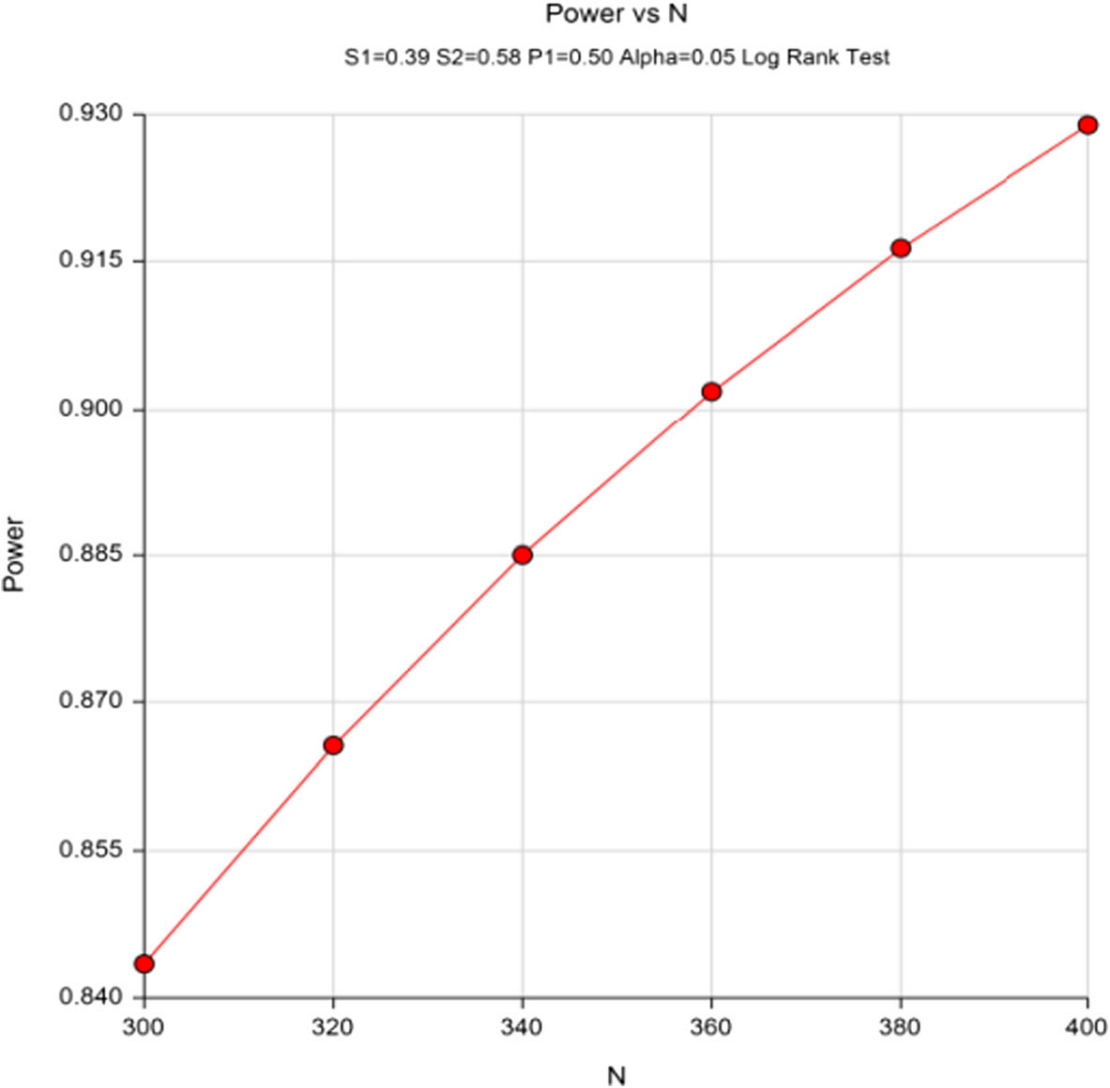
Power as a function of total sample size

A study by Cumming et al. [35] utilized a sample that is most similar to our target sample and also used daily calendars for fall ascertainment. On this basis, we assume that approximately 61% of control group participants will fall during 1 year of follow-up. For a meaningful reduction in fall risk, an intervention needs to demonstrate at least 30% reduction in falls [62, 63]. Because our target sample can be defined as “high risk,” and because our intervention can be defined as a high-intensity home modification, we project a 30% or greater reduction in fall risk at 12 months after treatment. Fig. 3 displays the power as a function of total sample size given a fall rate of 42% (event-free rate of 0.58) in the intervention group and a fall rate of 0.61 (event-free rate of 0.39) in the control group, with a 20% attrition rate. A two-sided log-rank test with an overall sample size of 300 participants (150 in each group) achieves 84% power at a 0.05 significance level to detect a difference of 0.19 between 0.39 and 0.58—the proportions without falls in the control and intervention groups, respectively.

## Discussion

This intervention is grounded in sound theory and evidence, allowing it to be effective for the target study population of underserved, difficult to reach older adults. The tailored approach of the study intervention is a strength in improving adherence, as each recommendation is individualized to be acceptable to the participant. Community engagement is a strength of this study. An advisory board has been established to counsel the research team in each phase of the study. The board has contributed to the development of the proposal, has actively participated in focus groups to prepare for the study including aspects of recruitment and intervention, and will continue to be involved throughout the study duration. The membership includes 2 to 3 service providers at the local, state, and national levels; 2 to 3 community leaders; and 2 to 5 older adults at high risk for falling. Finally, the design of the study allows for rapid dissemination of results and implementation of the intervention in a United States social services agency.

One limitation of this study is the inability to blind study participants and interventionists to group allocation. However, because the evaluators are blinded to group allocation, possible biases are reduced. Additionally, the control group lacks a time-equivalent social intervention. Although this is unlikely to affect the primary outcome of falls, there is a possibility that other variables of interest such as self-rated health may be improved in the intervention group as a result of social interaction with the therapists. Furthermore, participation in the study may increase awareness of falls for control group participants, causing them to seek resources or change behaviors to reduce the risk of falls.

Participants in the treatment group of this study benefit from home modifications that potentially improve safety and independence in the home. Participants also receive information on resources as well as training to increase awareness of fall risks and implement strategies for fall prevention in their homes. An unlikely risk of this study is the accidental disclosure of confidential participant information.

### Minimization of risks and confidentiality

REDCap is a secure, Web-based application designed to support data capture for research studies. REDCap servers are securely housed in an onsite, limited-access data center managed by the Division of Biostatistics at Washington University. All Web-based information transmission is encrypted. All data are stored on a private, firewall protected network. All users are given individual user IDs and passwords, and their access is restricted on a role-specific basis. REDCap was developed specifically around Health Insurance Portability and Accountability Act security guidelines and is implemented and maintained according to Washington University guidelines. Study data will be collected via tablet in the field and managed using REDCap electronic data-capture tools hosted at Washington University.

### Adverse event reporting and safety monitoring

All adverse event information is collected on an electronic Adverse Event (AE) Form. All AEs experienced by the participant from the start of intervention through the end of the study are to be reported.

Serious adverse events (SAEs) will be reported to the Human Research Protection Office (HRPO) in the following time frames: a) death – immediately; b) life-threatening – within 7 calendar days; c) all other SAEs – within 15 calendar days using the Electronic Serious Adverse Event Reporting System. Should there be a serious adverse event that occurs that increases the risks to the participants, the study will be stopped, an investigation will be conducted, and a findings report will be generated before the study is resumed.

### Dissemination

Results of the study will be submitted to peer-reviewed journals and disseminated at conferences on aging, occupational therapy, and public health. On publication of the study results, older adult participants will be invited to attend a community meeting during which the results of the study will be reported.

## Abbreviations

AAA: Area Agency on Aging
ADL: Activities of daily living
ED: Emergency department
FaB: Falls behavioral scale for older people
FES-I: Falls efficacy scale – international
GDS-SF: Geriatric depression scale short form
HARP: Home Hazard Removal Program
NAPIS: National Aging Program Information System
OARS: Older adults resources and services
OT: Occupational therapist/occupational therapy
POMA: Performance oriented mobility assessment
REDCap: Research electronic data capture
SBT: Short blessed test
SF-36: 36-Item short form survey
S-MAST-G: Short Michigan alcoholism screening test – geriatric version
SLAAA: St. Louis area agency on aging
WeHSA: Westmead home safety assessment

## References

[1] Tinetti ME, Speechley M, Ginter SF (1988). Risk factors for falls among elderly persons living in the community. N Engl J Med.

[2] Sattin RW, Rodriguez JG, DeVito CA, Wingo PA (1998). Home environmental hazards and the risk of fall injury events among community-dwelling older persons. Study to assess falls among the elderly (SAFE) group. J Am Geriatr Soc.

[3] Tinetti ME, Williams C (1997). Falls, injuries due to falls, and the risk of admission to a nursing home. N Engl J Med.

[4] Sattin RW (1992). Falls among older persons: a public health perspective. Annu Rev Public Health.

[5] Sterling DA, O'Connor JA, Bonadies J (2001). Geriatric falls: injury severity is high and disproportionate to mechanism. J Trauma Acute Care Surg.

[6] Alexander BH, Rivara FP, Wolf ME (1992). The cost and frequency of hospitalization for fall-related injuries in older adults. Am J Public Health.

[7] Tinetti ME, Baker, D.I., McAvay G., Claus, E.B., Garret, P., Gottschalk M., et al. A multifactorial intervention to reduce the risk of falling among elderly people living in the community. N Engl J Med 1994;331(13):821-827. 10.1056/NEJM199409293311301 8078528

[8] Arfken CL, Lach HW, Birge SJ, Miller JP (1994). The prevalence and correlates of fear of falling in elderly persons living in the community. Am J Public Health.

[9] National Council on Aging. Falls prevention: fact sheet. 2014. https://www.ncoa.org/resources/falls-prevention-fact-sheet/. Accessed 10 Aug 2014.

[10] Tromp AM, Pluijm SMF, Smit JH, Deeg DJH, Bouter LM, Lips P. Fall-risk screening test: A prospective study on predictors for falls in community-dwelling elderly. J Clin Epidemiol. 2001;54(8):837–44. 10.1016/s0895-4356(01)00349-3 11470394

[11] Englander F, Hodson TJ, Terregrossa RA (2006). The costs of fatal and nonfatal falls among older adults. Inj Prev..

[12] Mahoney J, Sager M, Johnson J (1994). Risk of falls after hospital discharge. J Am Geriatr Soc.

[13] Nachreiner NM, Findorff MJ, Wyman JF, McCarthy TC (2007). Circumstances and consequences of falls in community-dwelling older women. J Women's Health.

[14] Talbot L, Musiol R, Witham E, Metter EJ (2005). Falls in young, middle-aged and older community dwelling adults: perceived cause, environmental factors and injury. BMC Public Health.

[15] Gill TM, Williams CS, Tinetti ME (2000). Environmental hazards and the risk of nonsyncopal falls in the homes of community-living older persons. Med Care.

[16] Stevens JA, Corso PS, Finkelstein EA, Miller TR (2006). The costs of fatal and non-fatal falls among older adults. Inj Prev.

[17] Clemson L, Mackenzie L, Ballinger C, Close JC, Cumming RG (2008). Environmental interventions to prevent falls in community-dwelling older people: a meta-analysis of randomized trials. J Aging Health.

[18] Carter SE, Campbell EM, Sanson-Fisher RW, Redman S, Gillespie WJ (1997). Environmental hazards in the homes of older people. Age Ageing.

[19] Stevens M, Holman CDA, Bennett N (2001). Preventing falls in older people: impact of an intervention to reduce environmental hazards in the home. J Am Geriatr Soc.

[20] Nikolaus T (2003). Preventing falls in community-dwelling frail older people using a home intervention team (HIT): results from the randomized falls HIT trial. J Am Geriatr Soc.

[21] American Geriatrics Society: AGS/BGS clinical practice guidelines: prevention of falls in older persons. 2011. http://www.americangeriatrics.org/health_care_professionals/clinical_practice/clinical_guidelines_recommendations/prevention_of_falls_summary_of_recommendations. Accessed 18 Apr 2017.

[22] Gitlin LN, Winter L, Dennis MP, Corcoran M, Schinfeld S, Hauck WW (2006). A randomized trial of a multicomponent home intervention to reduce functional difficulties in older adults. J Am Geriatr Soc.

[23] Stark S, Landsbaum A, Palmer J, Somerville E, Morris J (2010). Home modifications improve daily activity performance of older adults. Can J Occup Ther.

[24] Israel BA, Schulz AJ, Parker EA, Becker AB (1998). Review of community-based research: assessing partnership approaches to improve public health. Annu Rev Public Health.

[25] Satcher D, Israel BA, Eng E, Schulz AJ, Parker EA (2005). Methods in community-based participatory research for health: Jossey-Bass.

[26] Minkler M, Wallerstein N (2003). Community-based participatory research for health.

[27] O'Toole TP, Aaron KF, Chin MH, Horowitz C, Tyson F (2003). Community-based participatory research. J Gen Intern Med.

[28] Branson RD, Davis K, Butler KL (2007). African Americans' participation in clinical research: importance, barriers, and solutions. Am J Surg.

[29] Levkoff S, Sanchez H (2003). Lessons learned about minority recruitment and retention from the centers on minority aging and health promotion. The Gerontologist.

[30] Loftin WA, Barnett SK, Bunn PS, Sullivan P (2005). Recruitment and retention of rural African Americans in diabetes research. Diab Educ.

[31] Linnan L, Steckler A (2002). Process evaluation for public health interventions and research: Jossey-Bass San Francisco, California.

[32] Show Me Falls Free Missouri Plan. An Action Plan for Preventing Falls Among Older Adults in the Community. In*.*: Missouri Department of Health and Senior Services; 2008.

[33] Close JCT, Lord SR, Antonova EJ, Martin M, Lensberg B, Taylor M, Hallen J, Kelly A (1999). Older people presenting to the emergency department after a fall: a population with substantial recurrent healthcare use. Emerg Med J.

[34] Albert M, McCaig LF, Ashman JJ (2013). NCHS data brief emergency department visits by persons aged 65 and over: United States, 2009–2010. In*.*, vol. 130.

[35] Cumming R, Thomas M, Szonyi G, Salkeld G, O'Neill E, Westbury C, Frampton G (1999). Home visits by an occupational therapist for assessment and modification of environmental hazards: a randomized trial of falls prevention. J Am Geriatr Soc.

[36] Katzman R, Brown T, Fuld P (1983). Validation of a short orientation memory concentration test of cognitive impairment. Am J Psychiatr.

[37] Edwards DF, Baum CM, Meisel M, Depke M, Williams J, Braford T, Morrow-Howell N, Morris JC (1999). Home-based multidisciplinary diagnosis and treatment of Inner-City elders with dementia. The Gerontologist.

[38] Williams MM, Meisel MM, Williams J, Morris JC (2011). An interdisciplinary outreach model of African American recruitment for Alzheimer's disease research. The Gerontologist.

[39] Harris PA, Taylor R, Thielke R, Payne J, Gonzalez N, Conde JG (2009). Research electronic data capture (REDCap)a metadata-driven methodology and workflow process for providing translational research informatics support. J Biomed Inform.

[40] Gitlin LN, Corcoran M, Winter L, Boyce A, Hauck WW (2001). A randomized, controlled trial of a home environmental intervention effect on efficacy and upset in caregivers and on daily function of persons with dementia. The Gerontologist.

[41] Clemson L, Roland M, Cumming RG (1997). Types of hazards in the homes of elderly people. Occup Ther J Res.

[42] Bodenheimer T, Lorig K, Holman H, Grumbach K (2002). Patient self-management of chronic disease in primary care. J Am Med Assoc.

[43] Miller WRR, S. (1991). Motivational interviewing: preparing people to change addictive behavior.

[44] Sarah E. Lamb ECJ-SKHCB. Development of a common outcome data set for fall injury prevention trials: the prevention of falls network Europe consensus. J Am Geriatr Soc 2005;53(9):1618-1622. 10.1111/j.1532-5415.2005.53455.x 16137297

[45] Kreuter MW, Oswald DL, Bull FC, Clark EM (2000). Are tailored health education materials always more effective than non-tailored materials?. Health Educ Res.

[46] Stark SL, Silianoff TJ, Kim HL, Conte JW, and Morris JC. Tailored Calendar Journals to Ascertain Falls Among Older Adults. OTJR: Occupation, Participation and Health. 2015;35(1):53–9. 10.1177/1539449214561764 PMC4391340 25866488

[47] Cella D, Riley W, Stone A, Rothrock N, Reeve B, Yount S, Amtmann D, Bode R, Buysse D, Choi S (2010). The patient-reported outcomes measurement information system(PROMIS) developed and tested its first wave of adult self-reported health outcome item banks: 2005-2008. J Clin Epidemiol.

[48] Fillenbaum GG, Smyer MA (1981). The development, validity, and reliability of the OARS multidimensional functional assessment questionnaire. J Gerontol.

[49] Yardley L, Beyer N, Hauer K, Kempen G, Piot-Ziegler C, Todd C (2005). Development and initial validation of the falls efficacy scale International (FES-I). Age Ageing.

[50] Ware JE, Sherbourne CD (1992). The MOS 36-ltem short-form health Survey (SF-36): I. Conceptual framework and item selection. Med Care.

[51] Rubenstein L, Josephson K (2006). Falls and their prevention in elderly people: what does the evidence show?. Med Clin North Am.

[52] Blow FC. Michigan Alcoholism Screening Test-Geriatric Version (MAST-G). 1991.

[53] Yesavage JA, Sheikh JI (1986). Geriatric depression scale (GDS) recent evidence and development of a shorter Version. Clin Gerontol.

[54] Thal LJ, Grundman M, Golden R (1986). A correlational analysis of the Blessed information-memory-concentration test and the mini-mental state exam. Neurology.

[55] Tinetti ME (1986). Performance-oriented assessment of mobility problems in elderly patients. J Am Geriatr Soc.

[56] Radomski MV, Trombly Latham C (eds.). Occupational Therapy for Physical Dysfunction, Sixth edn: Lippincott, Willams and Wilkins; 2008.

[57] Pelli DG, Robson JG, Wilkins AJ (1988). The design of a new letter chart for measuring contrast sensitivity. Clin Vision Sci.

[58] Clemson L, Cumming RG, Heard R (2003). The development of an assessment to evaluate behavioral factors associated with falling. Am J Occup Ther.

[59] Weersing VR, Rozenman M, Gonzalez A (2009). Core components of therapy in youth: do we know what to disseminate?. Behav Modif.

[60] Cumming RG, Thomas M, Szonyi G, Frampton G, Salkeld G, Clemson L (2001). Adherence to occupational therapist recommendations for home modifications for fall prevention. Am J Occup Ther.

[61] Lorig K SA, Ritter P, Gonzalez V, Laurent D, & Lynch J. Outcome measures for health education and other health care interventions. Thousand Oaks, CA: Sage Publications; 1996.

[62] Cumming RG (2002). Intervention strategies and risk-factor modification for falls prevention: a review of recent intervention studies. Clin Geriatr Med.

[63] Campbell AJ, Robertson MC, Gardner MM, Norton RN, Buchner DM (1999). Falls prevention over 2 years: a randomized controlled trial in women 80 years and older. Age Ageing.

